# Burnout and Stress in Forensic Science Jobs: A Systematic Review

**DOI:** 10.3390/healthcare12202032

**Published:** 2024-10-12

**Authors:** Claudia Lombardo, Emanuele Capasso, Giuseppe Li Rosi, Monica Salerno, Mario Chisari, Massimiliano Esposito, Lucio Di Mauro, Francesco Sessa

**Affiliations:** 1 Department of Biomedical and Biotechnological Sciences, University of Catania, 95121 Catania, Italy; claudia.lombardo@unict.it; 2 Department of Advanced Biomedical Science-Legal Medicine Section, University of Naples “Federico II”, 80131 Naples, Italy; emanuele.capasso@unina.it; 3 Faculty of Medicine and Surgery, “Kore” University of Enna, 94100 Enna, Italy; lirosigiose@gmail.com (G.L.R.); massimiliano.esposito@unikore.it (M.E.); 4 Department of Medical, Surgical and Advanced Technologies “G.F. Ingrassia”, University of Catania, 95121 Catania, Italy; monica.salerno@unict.it (M.S.); dr.luciodimauro@gmail.com (L.D.M.); 5 “Rodolico-San Marco” Hospital, Santa Sofia Street, 87, 95121 Catania, Italy; m.chisari@policlinico.unict.it

**Keywords:** burnout, stress, post-traumatic stress disorder (PTSD), forensic pathologists, forensic professionals, legal medicine

## Abstract

Background/Objectives. Burnout and occupational stress are significant issues among forensic professionals, impacting their well-being and job performance. This systematic review aims to provide an up-to-date overview of the occupational stress and burnout experienced by forensic personnel, exploring the profound and multifaceted impact on their physical, mental, professional, and interpersonal well-being. Methods. A systematic review was conducted following PRISMA guidelines using Scopus and WOS databases to search for articles published from 1 January 2000 to 31 August 2024. The search used keywords related to burnout and forensic professions. Inclusion criteria were original articles in English and French, while reviews, book chapters, editorials, and notes were excluded. A total of 10 studies were included after eliminating duplicates and excluding irrelevant articles. Results. The review identified seven key findings. (1) High levels of occupational stress and burnout among forensic personnel necessitate effective stress management strategies and resilience training; (2) autopsy technicians in Romania experience burnout and alexithymia, particularly related to traumatic events involving children, highlighting the need for specialized support systems; (3) disparities in burnout and post-traumatic stress disorder (PTSD) symptoms were observed in autopsy technicians and resident doctors, suggesting tailored mental health resources; (4) organizational factors, such as peer support and compensation satisfaction, significantly impact burnout and secondary traumatic stress (STS) among sexual assault nurse examiners; (5) burnout among forensic physicians, both in Romania and Egypt, is linked to personality traits, job satisfaction, and socio-demographic factors; (6) pathologists face a range of health issues, including musculoskeletal problems and psychological disorders, underscoring the need for industry-specific health measures; and (7) the lack of wellness resources for forensic professionals calls for improved mental health support and training. Conclusions. The findings highlight the pervasive issue of burnout and stress among forensic professionals globally. Addressing these challenges requires comprehensive stress management programs, tailored mental health resources, and organizational support. Future research should focus on developing and implementing effective interventions to enhance resilience and job satisfaction within this high-stress field.

## 1. Introduction

Healthcare is an operative field that generates great pressure among healthcare workers (HCWs), creating stress and burnout. Healthcare burnout can be described as a state of physical, mental, and emotional exhaustion caused by prolonged and excessive stress leading to a situation whereby healthcare professionals are unable to cope with the demands of their work. Consequently, subjects lose their morale and motivation, resulting in boredom [1,2,3]. Moreover, individuals affected by burnout may experience several issues, such as sleep disturbances [4,5].

Burnout comprises three categories: emotional exhaustion, which is a feeling of being drained; depersonalization, where there is a development of cynicism towards patients and colleagues; and reduced personal accomplishment, marked by ineffectiveness [6,7]. Stress, on the other hand, is the body’s way of dealing with change that is required for adaptation. HCWs who encounter these stressors experience pressures that include long working hours, life and death decision making, and the physical demands of their working conditions, and thus, they are at risk of developing cardiovascular diseases, musculoskeletal disorders, anxiety, and depression [8,9,10,11]. Numerous risk factors have been identified, including stress connected to competitiveness, inadequate coping mechanisms, excessive training, unfavorable group dynamics/peer relationships, and unfavorable interactions with leaders [12,13,14].

The causes of stress and burnout in HCWs are numerous and include the emotional toll of dealing with patients and paperwork as well as lack of autonomy in choosing working hours and scarcity of resources. The existing tendencies towards optimization of the work process and a reduction in costs in the sphere of healthcare intensify these issues and create circumstances that promote burnout and stress [15,16].

Stress and burnout issues, therefore, call for prevention strategies. Some of the measures that can be adopted by healthcare organizations include offering mental health services, creating a favorable working environment, offering professional development and training, and resolving staffing issues. On an individual level, healthcare professionals may be able to engage in health-promoting behaviors such as relaxation exercises, support seeking, and stress management [17,18].

While burnout and stress are widespread in healthcare in general, forensic professionals face unique challenges that can exacerbate these conditions. Their work often involves exposure to trauma and requires technical precision, placing them at higher risk for occupational stress. Indeed, among HCWs, forensic professionals are most affected by the conditions of burnout and stress in comparison to other specialists in the field of healthcare. Working in the field of forensics puts professionals in a position to deal with the consequences of criminal incidents for which they are at risk of developing vicarious trauma or secondary traumatic stress (STS) [19,20]. The workload and time pressures, the inherent nature of forensic work that is technical and detail oriented, the lack of control over one’s work, and administrative challenges add to their stress levels and risk of burnout. Moreover, based on a recent study, forensic medical staff workers are susceptible to psychological health symptoms, such as burnout or post-traumatic stress disorder (PTSD). Particularly, the personnel involved in the autopsy appeared to have higher emotional tiredness and indications of PTSD [21,22].

This systematic review aims to provide an up-to-date overview of the occupational stress and burnout experienced by forensic personnel, exploring the profound and multifaceted impact on their physical, mental, professional, and interpersonal well-being. This review also seeks to identify effective mitigation strategies to support the resilience and effectiveness of forensic professionals in their critical roles within the justice system. By understanding the definitions and clinical expressions of burnout and stress in this specialized field, this study aims to indicate interventions that can enhance the welfare of forensic professionals and, consequently, the quality of care they provide.

## 2. Materials and Methods

### 2.1. Study Design

A systematic review was conducted following the PRISMA (Preferred Reporting Items for Systematic Reviews and Meta-Analyses) guidelines to ensure a comprehensive and transparent assessment of the literature [23].

### 2.2. Data Sources and Search Strategy

The Scopus and Web of Science (WOS) databases were used to identify relevant studies published between 1 January 2000 and 31 August 2024. The search strategy included the following keyword combinations:

- (burnout) AND (autopsy)—twenty-five articles matched these keywords;
- (burnout) AND (post-mortem)—five articles matched these keywords;
- (burnout) AND (forensic pathologist)—two articles matched these keywords;
- (burnout) AND (medical examiner)—twenty-four articles matched these keywords;
- (burnout) AND (medico-legal doctor)—four articles matched these keywords;
- (burnout) AND (forensic examiner)—seventeen articles matched these keywords.

The numbers following each keyword combination indicate the initial number of studies identified through each search term (Figure S1).

### 2.3. Inclusion and Exclusion Criteria

The inclusion criteria for the review were as follows:

- Original articles;
- Articles written in English and French.

In order to focus on original research, the exclusion criteria included the following:

- Reviews (5);
- Book chapters (1);
- Editorials (2);
- Notes (2).

### 2.4. Quality Assessment and Data Extraction

An initial assessment of all articles was conducted by F.S., who evaluated the titles, abstracts, and full texts. M.S. then performed an independent reanalysis of the selected articles to ensure consistency. In cases of conflicting opinions regarding the inclusion of articles, E.C. was consulted for further evaluation and final decision making.

Cohen’s Kappa statistic [24] was used to assess the level of agreement between the studies, yielding a Kappa value of 0.90, which indicates a strong agreement among the included articles.

### 2.5. Characteristics of Eligible Studies

**Figure 1:**
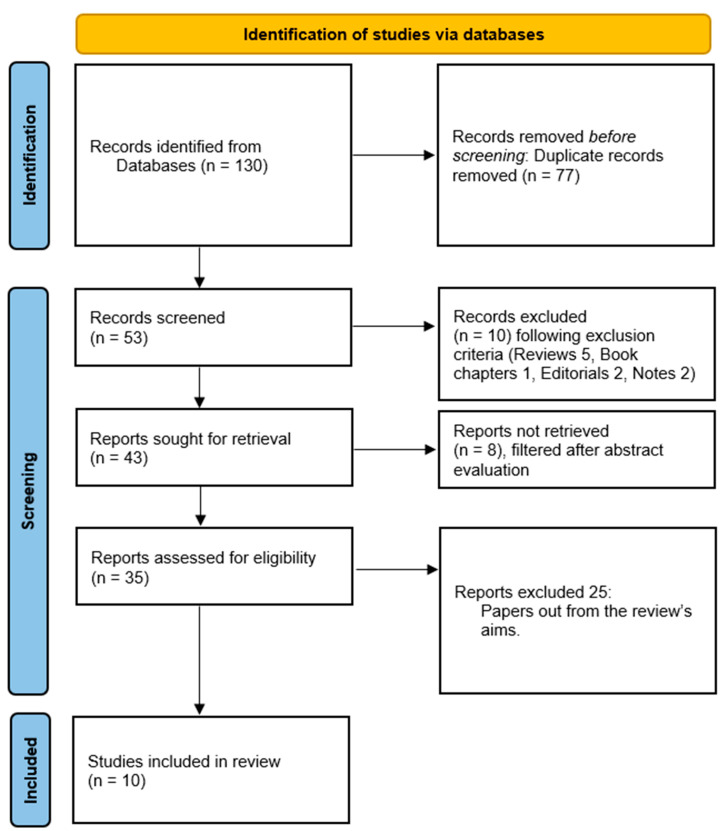
The PRISMA diagram for article selection: 10 articles were included after a rigorous selection process.

Out of an initial pool of 77 articles, 24 duplicates were removed. Additionally, 10 studies were excluded based on specific criteria. After a thorough evaluation process, a total of 10 articles met the inclusion criteria and were included in the systematic review (Figure 1).

This rigorous selection process ensured that the included studies provided valuable and relevant insights into the impact of burnout and stress on forensic professionals, forming the basis for the systematic review’s findings and conclusions.

## 3. Results

This section presents the findings from the literature review on burnout and stress among forensic professionals. Table 1 summarizes the main information for each selected study, methodologies, and main findings.

The systematic review highlights the pervasive issue of occupational stress and burnout among forensic professionals, underscoring the necessity for targeted interventions and support mechanisms within the field. High levels of stress and burnout are evident, as shown by Kelty et al. (2015) [25], who emphasized the importance of effective stress management strategies and organizational support to reduce burnout and turnover. Iorga et al. (2020) [26] found that autopsy technicians in Romania experienced low burnout but were significantly impacted by critical events, particularly involving children, suggesting further national-level research. Kömür et al. (2017) [21] identified higher emotional exhaustion and PTSD symptoms in autopsy technicians compared to resident doctors, highlighting intense work-related stressors. Townsend et al. (2009) [27] demonstrated that organizational factors, including peer support and satisfaction with compensation, mitigated STS and burnout among sexual assault nurse examiners. Iorga et al. (2016) [22] and Goldstein et al. (2021) [28] both stressed the need for enhanced mental health support and wellness resources for forensic professionals, emphasizing the profession’s impact on personal lives and the inadequacy of current training programs. Sehsah et al. (2021) [29] noted high burnout levels among Egyptian forensic physicians with significant predictors, including frequent stressful duties and being female. Levin et al. (2021) [30] found that organizational efforts significantly reduced STS and burnout while increasing compassion satisfaction among forensic science professionals. Dervaux et al. (2020) [31] revealed high rates of musculoskeletal and psychological issues among French pathologists, pointing to a lack of adequate medical follow-up and training on chemical risks. Almazrouei et al. (2021) [32] identified significant stress factors for forensic examiners, including managerial issues and case backlogs, with higher stress levels reported by female examiners and those with more experience. Collectively, these studies emphasize the urgent need for comprehensive support systems, targeted interventions, and organizational reforms to enhance the well-being and performance of forensic professionals.

## 4. Discussion

This literature review provides a comprehensive overview of the multifaceted nature of stress and burnout among forensic professionals, highlighting the critical need for tailored interventions, organizational support, and industry-specific health and safety measures to enhance their well-being and performance. Comparing these findings with the international literature reveals both commonalities and unique challenges faced by forensic professionals globally. The issue of occupational stress and burnout among forensic personnel is well documented internationally, with studies from various countries consistently reporting high levels of stress and burnout in forensic professions [33]. During the recent pandemic, stress factors significantly increased due to the risks of infection associated with forensic activities. Indeed, considering every contact could potentially result in infection, forensic personnel experienced heightened distress events, both during crime scene investigations and medico-legal activities [34,35]. Another crucial aspect is related to autopsies. In the initial phase of the COVID-19 outbreak, the scientific community banned autopsies to prevent virus outbreaks [36,37]. This decision may have contributed to delays in understanding the virus’s behavior, considering that autopsies should be regarded as the gold standard for evaluating unknown infections [38]. Therefore, the benefits to the pathologist must be weighed against the broader public interest, considering that, as later confirmed, the body is usually considered safe [39,40,41].

Research has similarly emphasized the importance of resilience training and stress management as key strategies for enhancing job performance and well-being among forensic professionals [42]. Resilience training has been widely implemented across various healthcare sectors, particularly in high-stress environments such as emergency medicine, nursing, and critical care. In these fields, resilience programs often focus on stress reduction, mindfulness, cognitive behavioral techniques, and building emotional regulation skills to help professionals cope with chronic stress and prevent burnout. For example, in emergency care settings, resilience programs that incorporate mindfulness training, peer support groups, and professional development workshops have demonstrated significant improvements in reducing burnout and enhancing job satisfaction [43,44]. Additionally, in nursing, resilience training often includes components such as team-building exercises, mentorship, and structured debriefing sessions after critical events, which have been shown to bolster emotional resilience and promote well-being [45,46]. For forensic professionals, similar approaches could be adopted but tailored to the unique stressors of their work environment. Resilience training for this group could focus on emotional regulation techniques specific to the trauma encountered in forensic settings, including coping with vicarious trauma and secondary traumatic stress. Structured debriefings after emotionally challenging cases, such as those involving autopsies or crime scene investigations, may provide a much-needed outlet for emotional processing. In addition, resilience programs could include specialized training on managing the technical and detail-oriented nature of forensic work, where errors are less tolerated and stress levels may be amplified. Given that forensic professionals often face isolation in their work, peer support networks could be particularly beneficial, providing an opportunity for shared experiences and collective coping strategies. This suggests that the findings of this review align with broader international trends, underscoring the universal need for effective stress management programs. The presence of burnout and alexithymia among autopsy technicians in Romania, particularly related to critical events involving children, mirrors findings in other countries where autopsy technicians and professionals in similar roles experience significant emotional tolls. For instance, several studies have documented similar challenges, where the nature of the work leads to high emotional exhaustion and impacts mental health [21,43]. These studies advocate for the implementation of debriefing sessions and access to psychological support, consistent with the recommendations of this review. The disparities in burnout and PTSD symptoms between autopsy technicians and resident doctors found in this review are also reflected in the international literature. In Japan and Germany, for example, research has shown that the intensity and nature of exposure to traumatic events significantly influence burnout levels among different forensic roles [44,45,46]. This supports the call for tailored mental health resources to meet the specific needs of various subgroups within the forensic field. The impact of organizational factors on STS and burnout among SANE nurses highlighted in this review is corroborated by studies from the United States and Sweden. These studies emphasize the role of organizational support, peer relationships, and adequate compensation in mitigating stress and burnout [47,48]. The findings suggest that organizational interventions are critical for improving the working conditions of SANE nurses and reducing their stress levels. The focus on individual differences in how forensic physicians experience and cope with burnout is a theme also present in the international literature. Research in Italy and Brazil has highlighted the role of personality traits, job satisfaction, and socio-demographic factors in influencing burnout among forensic physicians [49,50]. This indicates that personalized interventions considering these factors are necessary to effectively address burnout in this professional group. The survey findings on health issues among pathologists, including musculoskeletal and visual problems, are consistent with studies from countries such as Switzerland and Spain. These studies report similar occupational health risks and advocate for enhanced medical follow-up and ergonomic training [51,52]. This suggests that the need for industry-specific health and safety measures is a global concern. The lack of wellness resources among forensic professionals, as discussed in this review, is echoed in international studies. Research from Norway and New Zealand highlights the critical need for integrating mental health support within education and training programs and fostering a supportive professional community [53,54,55]. This approach is essential for normalizing mental health discussions and providing necessary support. The investigation into STS, burnout, and compassion satisfaction in forensic science professionals underscores the significant role of organizational efforts. Similar findings are reported in studies from France and India, which emphasize that institutional support can significantly reduce stress and burnout while enhancing compassion satisfaction [30,56,57].

**Figure 2:**
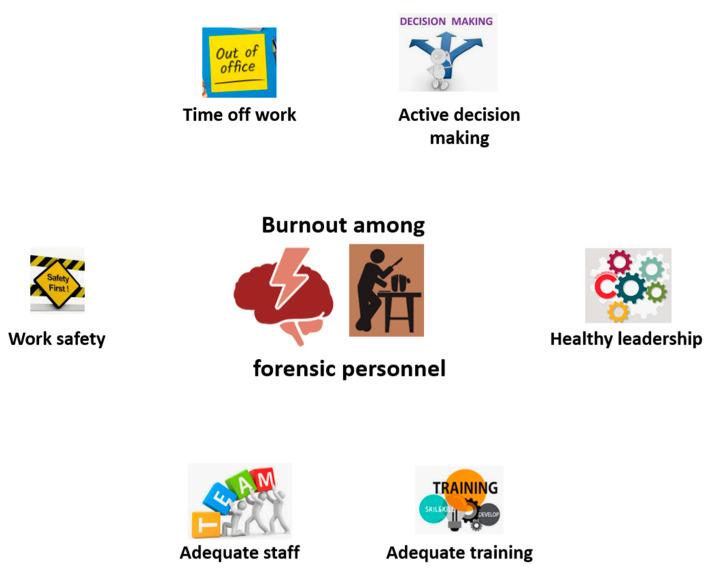
Key strategies to prevent burnout among forensic personnel: a comprehensive approach focusing on work–life balance, decision-making inclusion, leadership support, adequate staffing, and prioritizing safety and health.

Based on the findings of this literature review, as summarized in Figure 2, burnout among forensic personnel can be effectively prevented by implementing a comprehensive set of strategies focused on well-being and support. Key measures include modeling and encouraging the use of time off, which allows workers to recharge and maintain a healthy work–life balance. Involving forensic workers in decision-making processes ensures that they feel valued and heard, fostering a sense of ownership and satisfaction. Assigning a senior leader to oversee staff well-being emphasizes the organization’s commitment to their mental and physical health. Ensuring adequate staffing levels is crucial to avoid overburdening employees, while training supervisors to provide support creates a positive and empathetic work environment. Additionally, prioritizing worker safety and health is essential to protect forensic personnel from the unique challenges and stresses they face in their roles.

This underscores the importance of proactive organizational policies and support systems. Finally, the scarcity of research on workplace stress for forensic examiners identified in this review reveals a gap that is also noted in the international literature. Studies from other countries highlight significant stress factors, such as management, supervisors, and case backlogs, with demographic factors and career stage influencing stress perception [29,32,58]. This calls for targeted interventions that consider these variables.

Moreover, future research should evaluate several characteristics that are usually not evaluated. For example, the genetic substrate may negatively impact the insurgence of burnout. Indeed, serotonin (5-HT) neurotransmission plays a major role in controlling central nervous system activity and affects a wide range of physiological and psychological functions, such as individual variances in personality characteristics. A polymorphism is present in the promoter region of the SLC6A4 gene that encodes for serotonin transporter protein (5-HTT). Strong evidence has been found in several studies linking the 5-HTTLPR short allele to neuroticism, which is characterized by a propensity for negative emotionality, such as anxiety and sadness [12,59,60]. In this context, the evaluation of this polymorphism could be performed in order to disclose if the insurgence of burnout occurs more frequently in subjects with the short variant.

Another interesting research field to understand how burnout evolves concerns the role of neurobiological factors that are involved in the regulation of wakefulness, stress responses, motivation, and sleep. Particularly, the dysregulation of the orexin system in response to chronic stress may lead to physical and emotional exhaustion, impaired cognitive function, and sleep disturbances that characterize burnout [61,62,63,64]. Chronic stress is closely related to dysregulation of the hypothalamic–pituitary–adrenal (HPA) axis, the body’s central stress response system. Persistent stress can lead to alterations in cortisol levels, which affect energy balance, mood, and overall well-being [61,65,66]. The orexin system is highly responsive to stress, and it has been suggested that chronic stress may lead to dysregulation in orexin signaling. This disruption could contribute to the symptoms observed in burnout, particularly chronic fatigue, emotional exhaustion, and impaired cognitive function. In this way, it could be important to conduct future research checking the orexin plasma level in the forensic professional in relation to its specific activities.

## 5. Conclusions

In conclusion, this review provides a comprehensive understanding of stress and burnout among forensic professionals, revealing that these issues are global and multifaceted, providing several ideas for future research. The comparison with the international literature underscores the universal nature of these challenges and the critical need for tailored interventions, organizational support, and industry-specific health and safety measures. Some of these issues can be solved in parallel, which may result in better job satisfaction, decreased turnover rate, and enhanced mental health among forensic personnel around the globe.

We recommend the immediate implementation of structured stress management programs, resilience training, and organizational interventions in forensic workplaces to address these urgent concerns. Healthcare and forensic organizations should also prioritize developing mental health services that are integrated into both training curricula and ongoing professional development programs. Specifically, it is important to monitor the mental health of each forensic professional.

Further research should be directed toward creating and assessing the efficacy of preventive measures such as resilience training, proper organizational support, and comprehensive health and safety programs concerning diverse subgroups of forensic professionals. Future studies should also explore innovative approaches for early detection of burnout, including genetic predispositions and neurobiological markers, to better understand and mitigate its onset. By taking these proactive steps, organizations can ensure a healthier, more supportive work environment that enhances both individual well-being and professional performance. In addition, promoting a supportive professional culture and implementing mental health services into the curricula and professional development are crucial measures for preventing stress and burnout in this crucial field.

## Figures and Tables

**Table 1:** In this table, the main information for each selected study, methodologies, and main findings are reported.

First Author; Year	Title	Methodology	Main Findings
Kelty et al., 2015 [25].	No Burnout at this Coal-Face: Managing Occupational Stress in Forensic Personnel and the Implications for Forensic and Criminal Justice Agencies.	Interview with crime scene examiners (CSEs).	The qualitative data highlighted effective coping mechanisms among high-performing CSEs, while the quantitative results identified key attributes for recruitment and professional development. The study emphasized the need for organizational support programs to reduce burnout and turnover.
Iorga et al., 2020 [26].	Burnout, alexithymia and job satisfaction in autopsy technicians.	Self-administered questionnaire using three psychological instruments to assess burnout, job satisfaction, and alexithymia; statistical analysis with SPSS version 21.	Autopsy technicians in Romania displayed low levels of burnout and high job satisfaction. Critical events, particularly those involving children, impacted their lives and job satisfaction. The study underscored the need to address job-related events affecting alexithymia.
Kömür et al., 2017 [21].	PTSD and Burnout Symptoms in Forensic Doctors and Staff in a Mortuary.	Data collection from 142 mortuary staff using standardized questionnaires; statistical analysis with ANOVA, Tukey tests, χ2 tests, and correlation analysis using SPSS.	The study found higher emotional exhaustion and PTSD symptoms among autopsy technicians compared to resident doctors, highlighting significant work-related stressors.
Townsend et al., 2009 [27].	Organizational correlates of STS and burnout among sexual assault nurse examiners.	Data collection over a specific period, comprehensive sampling, interviews, and statistical analyses.	Organizational variables significantly predicted STS and burnout among sexual assault *nurse* examiner (SANE) nurses. Protective factors included peer support, satisfaction with compensation, SANE-only facilities, age, and education. The study recommended reevaluating program missions, particularly regarding prosecution orientation.
Iorga et al., 2016 [22].	The burnout syndrome of forensic pathologists. The influences of personality traits, job satisfaction and environmental factors.	Data from 37 forensic physicians using three questionnaires (MBI, BFI, JSS); analysis with SPSS.	Forensic physicians employed coping strategies and sought professional help. The study found associations between job satisfaction, personality traits, and burnout dimensions.
Goldstein et al., 2021 [28].	Self-reported levels of occupational stress and wellness in forensic practitioners: Implications for the education and training of the forensic workforce.	Survey of forensic practitioners with a 22 question survey; statistical analysis using chi-square cross-tabulations and correlation analyses.	Forensic professionals reported high burnout levels and inadequate wellness resources. Mental health issues were insufficiently addressed during training. The study highlighted significant trends related to vicarious trauma.
Sehsah et al., 2021 [29].	Work burnout and coping strategies among Egyptian forensic physicians: a national study.	Cross-sectional study with self-administered questionnaires; data analysis with logistic regression and correlation analysis.	High burnout levels were prevalent among Egyptian forensic physicians. Significant predictors included being a forensic examiner, frequent exposure to stressful duties, and being female. Adaptive coping was the most common strategy.
Levin et al., 2021 [30].	STS, burnout, compassion satisfaction, and perceived organizational trauma readiness in forensic science professionals.	Online surveys using modified ProQOL and VT-ORG questionnaires; statistical analyses and onsite visits for anonymity.	Field-based forensic professionals experienced higher STS levels compared to laboratory-based professionals. Organizational support efforts were linked to lower STS and burnout and higher compassion satisfaction. The study emphasized the need for organizational interventions to address stress and trauma.
Dervaux et al., 2020 [31].	Pathologist occupational hazards: Results of a survey for the French case.	Anonymous online questionnaire for French pathologists; statistical analysis of responses.	Pathologists reported a high prevalence of musculoskeletal and visual disorders, injuries during sampling, and moderate psychological issues, such as depression and burnout. Despite overall job satisfaction, there was a lack of medical follow-up, ergonomic advice, and training on chemical risks.
Almazrouei et al., 2021 [32].	Stress and support in the workplace: The perspective of forensic examiners.	Survey of 41 forensic examiners using a questionnaire with a seven-point Likert scale.	Significant stress factors included managerial and supervisory issues and case backlogs. Female examiners reported higher stress levels than males, and those with 11–15 years of experience felt more pressure than those with 7–10 years. The study highlighted the high-stress environment and sources of stress among forensic examiners.

## Data Availability

No new data were created or analyzed in this study.

## Supplementary Materials

The following supporting information can be downloaded at: https://www.mdpi.com/article/10.3390/healthcare12202032/s1.
