# Supplementary figures and images for: Burnout and Stress in Forensic Science Jobs: A Systematic Review
Source: Healthcare (Basel). 2024 Oct 12;12(20):2032. doi: 10.3390/healthcare12202032 (PMC11506976; doi:10.3390/healthcare12202032)

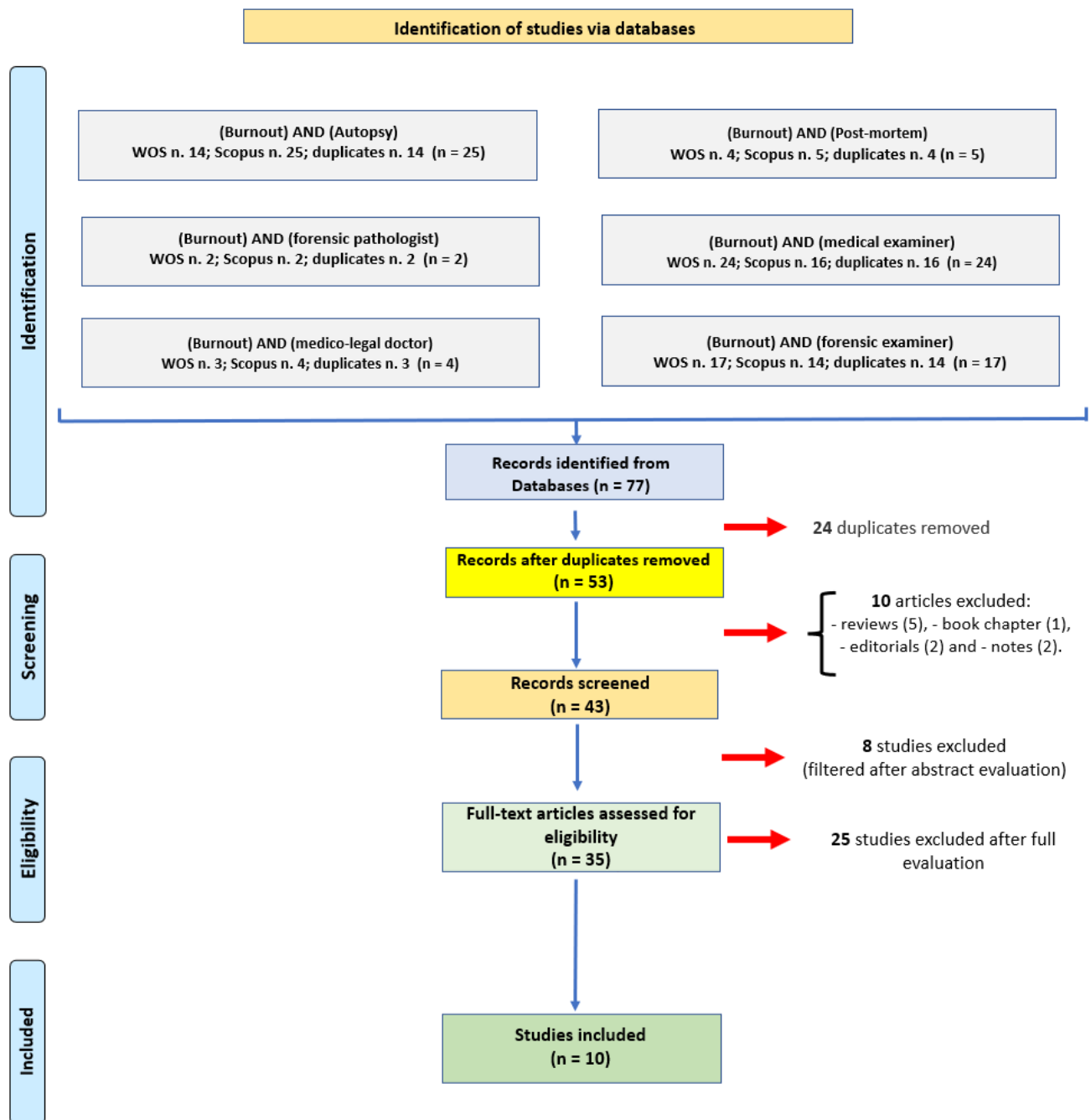

**Figure S1.** This is a figure summarized the article's selection process.

Supplement: Supplementary file 1 [file healthcare-12-02032-s001.zip › healthcare-3225707-supplementary.pdf]
